# Reducing bias in population and landscape genetic inferences: the effects of sampling related individuals and multiple life stages

**DOI:** 10.7717/peerj.1813

**Published:** 2016-03-14

**Authors:** William Peterman, Emily R. Brocato, Raymond D. Semlitsch, Lori S. Eggert

**Affiliations:** 1 School of Environment and Natural Resources, The Ohio State University, Columbus, OH, United States; 2 Division of Biological Sciences, University of Missouri - Columbia, Columbia, MO, United States

**Keywords:** *Ambystoma*, Sibship, Complex life cycle, Amphibian, Landscape genetics, Microsatellite, Mixing tissue samples, Genetic sampling, Population genetics, Salamander

## Abstract

In population or landscape genetics studies, an unbiased sampling scheme is essential for generating accurate results, but logistics may lead to deviations from the sample design. Such deviations may come in the form of sampling multiple life stages. Presently, it is largely unknown what effect sampling different life stages can have on population or landscape genetic inference, or how mixing life stages can affect the parameters being measured. Additionally, the removal of siblings from a data set is considered best-practice, but direct comparisons of inferences made with and without siblings are limited. In this study, we sampled embryos, larvae, and adult *Ambystoma maculatum* from five ponds in Missouri, and analyzed them at 15 microsatellite loci. We calculated allelic richness, heterozygosity and effective population sizes for each life stage at each pond and tested for genetic differentiation (*F*_ST_ and *D*_*C*_) and isolation-by-distance (IBD) among ponds. We tested for differences in each of these measures between life stages, and in a pooled population of all life stages. All calculations were done with and without sibling pairs to assess the effect of sibling removal. We also assessed the effect of reducing the number of microsatellites used to make inference. No statistically significant differences were found among ponds or life stages for any of the population genetic measures, but patterns of IBD differed among life stages. There was significant IBD when using adult samples, but tests using embryos, larvae, or a combination of the three life stages were not significant. We found that increasing the ratio of larval or embryo samples in the analysis of genetic distance weakened the IBD relationship, and when using *D*_*C*_, the IBD was no longer significant when larvae and embryos exceeded 60% of the population sample. Further, power to detect an IBD relationship was reduced when fewer microsatellites were used in the analysis.

## Introduction

An overarching goal of any study is to obtain accurate, unbiased estimates of the parameters of interest. In population and landscape genetics, it is often recommended that 25–30 individuals be sampled from each population (Hale, Burg & Steeves, 2012). For many species or systems, it is often easiest to meet these requirements by sampling early life stages (e.g., eggs or larvae) that can be found in abundance within a discrete area. However, many organisms experience extremely high mortality in these early life stages. Most amphibian, fish and insect species are characterized by Type III survivorship, in which a majority of young individuals will die before reaching sexual maturity, and the genetic characteristics of these life stages may differ from the few surviving adults due to the decrease in population size (Frankham, 1996). While it may often be assumed that selection pressures that reduce population size act uniformly and randomly, selection may differentially affect individuals. For example, numerous studies have assessed the role of inbreeding and heterozygosity on individual fitness (e.g., Balloux, Amos & Coulson, 2004; Ficetola et al., 2011; Harrison et al., 2011; Slate et al., 2004). Both of these population genetic attributes are particularly relevant in species of conservation concern, which often exist in small or isolated populations. Given the interaction between selection pressures and genetic diversity, it is not unreasonable to believe that population genetic measures may differ depending on the age or life stage of the sampled cohort.

Despite the potential problems with sampling different life stages, it is not uncommon for population or landscape genetic studies to combine samples from different cohorts or life stages, either because of convenience or necessity. Early life stages are often sampled because they are accessible, abundant, and cost-effective (Heyer et al., 1994). In amphibians, the extreme decline in individuals from early life stages to adults has been well-documented. Peterson et al. (1991), found a pre-metamorphic mortality rate of 99% in ringed salamanders (*Ambystoma annulatum*), Shoop (1974) found that pre-metamorphic mortality rates ranged from 87–99% in spotted salamanders (*A. maculatum*), and Berven (1990) recorded pre-metamorphic mortality rates ranging from 97–99% in wood frogs (*Rana sylvatica*). The drastic decline in abundance can also be seen in fish and insects. Dahlberg (1979) found a mortality rate of >99% in the eggs of many fish species, while a study of the southern green stink bug (*Nezara viridula*) found mortality rates to be as high as 96% (Kiritani & Nakasuji, 1967). As such, when early life stages are sampled to make inferences about the adult population, biased conclusions may result (Allendorf & Phelps, 1981; Goldberg & Waits, 2010). Obtaining unbiased estimates of genetic diversity is particularly critical for management and conservation of species.

Sampling animals from the field is often opportunistic due to the availability of the target species. Environmental factors, stochastic events, or the timing of offspring can alter when a life stage becomes available, if it can be found at all (Mullins, Pierce & Gutzwiller, 2004). In these cases, researchers often need to stray away from their sampling scheme and target life stage, and collect other life stages to reconcile the sample size gap (e.g., Lee-Yaw et al., 2009; Richardson, 2012). Despite the relative commonness of these sampling realities, the effect of mixing life stages in population and landscape genetic analyses has not been explicitly addressed. The sampling of full siblings has been shown to affect the estimates of population genetic parameters (Goldberg & Waits, 2010). When sampling amphibians, field researchers have the highest probability of collecting sibling pairs within larvae (Goldberg & Waits, 2010); related larvae are often spatially clustered, and samples collected at a specific location may be biased towards a single family group (Hansen, Nielsen & Mensberg, 1997). If researchers are unaware that family groups are being sampled, the genetic structure of the family could be misinterpreted as population structure within the panmictic population (Anderson & Dunham, 2008). To prevent misinterpretations and avoid biased population genetic parameter estimates, it has been suggested that samples be screened prior to analysis, and full siblings removed (Goldberg & Waits, 2010).

To date, only Goldberg & Waits (2010) have empirically tested the effects of sampling different life stages in amphibians (one frog and one salamander species) and quantified the importance of removing full siblings prior to analysis. The primary objective of this study was to determine the effects of pooling different life stages on population and landscape genetic inferences. Additionally, we sought to extend the findings of Goldberg & Waits (2010) to determine the effect of sampling three life stages in amphibians: adults, embryos, and larvae, on population and landscape genetic inferences. We assessed these objectives both with and without full-sibling pairs present in the data set, and also assessed how inferences are affected by reducing the number of microsatellites used for analysis. We predicted that the random mixing of life stages would result in genetic parameter estimates that did not differ from estimates of individual life stages. However, we predicted that there would be significant biases present when sampling different life stages as certain alleles are likely to be over-represented in the embryonic and larval life stages. Finally, we predicted that the removal of siblings from the data set would significantly alter population and landscape genetic estimates by increasing average allelic richness and heterozygosity within a sampled population, as well as increasing the average genetic differentiation among populations.

## Materials and Methods

### Ethics statement

This research was conducted in compliance with all laws and regulations for the state of Missouri and the USA, and was conducted under Missouri Wildlife Collector’s permit 15584. Sampling methods were approved by the University of Missouri Animal Care and Use Committee (Protocol 7403).

### Data availability

All data and code used in this study can be accessed from Figshare at https://dx.doi.org/10.6084/m9.figshare.1621318.v2.

### Literature review

To determine how researchers are currently collecting tissue samples from amphibians with complex life cycles, we conducted a literature search of the Scopus database of population and landscape genetic studies of amphibians. We used the search terms “amphibia*” (occurring in the title, abstract, or keywords), “microsatellite*” (occurring in all fields), and NOT “reptil*” (occurring in the title, abstract, keywords) and limited the search to findings from Molecular Ecology, Conservation Genetics, Heredity, Biological Journal of the Linnean Society, Amphibia-Reptilia, Animal Conservation, Molecular Ecology Resources, Evolution, Plos One, or Journal of Zoology published through December 2012. For each study, we determined if different life stages were sampled and if the study gave an indication as to whether sampling multiple life stages influenced analysis or inferences made from the data.

**Figure 1:**
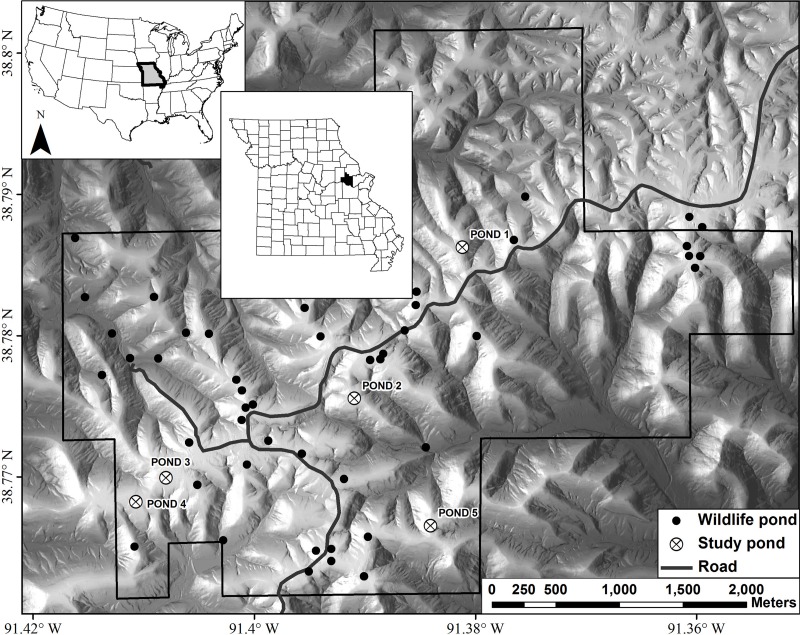
Map of Daniel Boone Conservation Area depicting the locations of the five ponds used in this study. Wildlife ponds are ponds readily used by amphibians, such as *Ambystoma maculatum*, for reproduction.

### Sampling

Our study was conducted at Daniel Boone Conservation Area (DBCA), in Warren County, Missouri, USA (Fig. 1). This 1,424 ha area is situated on the upper Ozark Plateau physiographic region and is characterized by mature (80–100 years old) second-growth forest with an overstory dominated by oak (*Quercus* spp.) and hickory (*Carya* spp.), with varying amounts of sugar maple (*Acer saccharum*) and red cedar (*Juniperus virginiana*) in the understory (Semlitsch et al., 2009). There are >40 fishless manmade ponds that are, on average, separated by 2,000 m (246–3,900 m) (Peterman et al., 2013b). Only man-made ponds are known to still exist on the DBCA landscape. We sampled adults, embryos, and larvae of *Ambystoma maculatum* (spotted salamander) from five ponds at DBCA (Fig. 1). Each of these ponds have been the focus of previous amphibian research at DBCA (e.g., Hocking et al., 2008; Semlitsch et al., 2014), and have similar surface area (160–330 m^2^), depth (<1.2 m), age (27–47 yrs), and permanent hydroperiod. We sought to collect 25 adult and embryo samples and 30 larval samples from each pond. Adult salamanders were captured in mesh funnel traps placed in breeding ponds in March 2013, and tissue samples were obtained by removing 0.5 cm of tail tissue. Following oviposition, we sampled embryos by collecting a single embryo per clutch in April 2013. In June 2013 larvae were captured with dip nets, and to minimize the sampling of siblings, we collected larvae from the entire perimeter of each pond. Upon collection in the field, each tissue sample was placed in 95% ethanol and stored at −20 °C until DNA extraction.

### Lab techniques

DNA was extracted from tissue using chelex-based resin (InstaGene; BioRad, Hercules, CA, USA). Approximately 2.5 mm × 2.5 mm of tissue was finely chopped with a sterile razor and was incubated at 60 °C for 2 hrs in 250 µL of InstaGene, vortexed, incubated for 20 min at 100 °C, then vortexed again. Following centrifugation, a 100 µL aliquot was removed and used as template DNA and the remainder was kept at −20 °C (Peterman et al., 2012). Nineteen tetra-nucleotide microsatellite loci were amplified using PCR; primers were fluorescently 5′ labeled with FAM, NED, VIC, and PET and arranged into two multiplex reactions (Peterman et al., 2013a). Negative controls were included in all reactions to detect contamination of reagents. Amplification products were sized on an ABI 3730xl DNA Analyzer (Applied Biosystems, Foster City, CA, USA) using Liz 600 size standard at the University of Missouri DNA Core Facility, and results were scored using GENEMARKER (v.1.97; Softgenetics, State College, PA, USA).

### Differences among life stages

Before proceeding with analyses we created a data set free of full sibling pairs using COLONY (Wang, 2012). For our COLONY analyses, both male and female mating were set to polygamous without inbreeding. We conducted a long run with full likelihood and high precision and did not include a sibship prior. We excluded siblings from the analysis such that all sites only had one individual per family group. Values for *F*_ST_ and allelic richness (rarefied to our smallest sample) were calculated with the R package *hierfstat* (Goudet, 2013), observed heterozygosity and chord distance (*D*_*C*_) were calculated with the R package *adegenet* (Jombart & Ahmed, 2011), and effective population size estimates (*N*_*e*_) were made using the linkage disequilibrium method implemented in COLONY (Wang, 2012). The proportion of siblings removed from each life stage at each pond was also calculated. To determine the effect of sibling removal, we also calculated summary statistics (*H*_*o*_, *A*_*r*_, *F*_ST_, *D*_*C*_) for each life stage with siblings present. All population genetic measures were compared among life stages and between estimates made with and without siblings using analysis of variance (ANOVA) and paired *t*-tests. Due to small sample sizes, we bootstrapped our ANOVA analyses and conducted permutation *t*-tests to more robustly assess differences among life stages and removal of siblings.

### Effect of mixing life stages

Prior to pooling life stages together, we conducted a second removal of related individuals using the COLONY settings described above (Wang, 2012). Specifically, we found and removed parent–offspring and embryo-larvae sibling pairs within each pond. All unrelated individuals of all life stages were pooled by pond of origin to make five mixed-tissue populations. From these populations, we randomly sampled 25 individuals using the R package *hierfstat* (Goudet, 2013) in R (R Core Team, 2013). This bootstrap resampling procedure was repeated 1,000 times (both with and without siblings), and the mean and 95% confidence intervals were calculated for *H*_*o*_, *A*_*r*_, *F*_ST_, and *D*_*C*_.

### Isolation-by-distance analysis

For the isolation-by-distance (IBD) analysis we conducted simple Mantel tests correlating genetic distance with Euclidean distance between ponds. This test was repeated for all life stages, with and without siblings, and significance was assessed using 100,000 permutations using the R package *ecodist* (Goslee & Urban, 2007). We tested for IBD in the mixed sample population, and calculated the mean and 95% confidence interval for both the Mantel *r* correlation statistic and the associated *P*-value based on the 1,000 bootstrap iterations. Because we found a significant IBD relationship when using adult-only tissue samples (see ‘Results’), we further assessed how the IBD relationship changed with the inclusion of larval and embryo samples. For this analysis, we varied the proportion of larval and embryo samples included with our adult samples. This was assessed at proportions ranging from 0 (no larval or embryo samples) to 1 (only larval and embryo samples) at increments of 0.05. At each increment, we assessed the mean and 95% confidence intervals of the Mantel *r* and the corresponding *P*-value based on 1,000 bootstrap samples of the data. We used the data set without siblings for this analysis and sampled each population to the minimum adult sample size (*n* = 18).

### Number of microsatellite loci

Concurrent with assessing the effects of mixing life stages, we also assessed the effects of reducing the number of microsatellites used in an analysis. Within the bootstrapping procedure for assessing the proportion of larval and embryo samples described above, we sub-sampled our microsatellite data set to include either 5, 10, or all 15 of the microsatellites. At each bootstrap iteration at each mixture proportion, microsatellites were randomly chosen to calculate Mantel *r* and the corresponding *P*-value. We also calculated the observed heterozygosity and allelic richness at each mixture proportion and for each level of microsatellite subsampling. These estimates were averaged over all populations sampled.

## Results

### Literature review

We found that 20 out of 95 (21%) of studies meeting our search criteria on Scopus conducted population or landscape genetic analyses of amphibian species using mixed tissue sampling (searched on 13 February 2016). Five of these studies stated that one life stage was sampled only when the target life stage was not available (Beebee & Rowe, 2000; Lee-Yaw et al., 2009; Lee-Yaw, Irwin & Green, 2008; Munwes et al., 2010; Richardson, 2012). None of these studies made attempts to check or correct for the effects of mixing life stages in their analyses, although it was common for siblings to be removed prior to analysis.

### Sample summary

We collected 24–25 adults and 19–27 embryos from each of the five ponds, and 29–36 larvae from three of the five ponds (Supplemental Information 1). We were unable to sample larvae from two of the ponds due to high embryo mortality. Of the original 19 screened primers, two loci (*Am_13*, *Am_60*) were not polymorphic, and two loci (*Am_33*, *Am_43*) showed very little polymorphism and deviated significantly from expected heterozygosity values under Hardy Weinberg equilibrium (HWE). These four loci were removed from the dataset and all population genetic statistics were calculated using the remaining 15 loci (Supplemental Information 1). No other loci or populations deviated from Hardy-Weinberg equilibrium or were significantly linked. Overall, we had <0.5% missing data.

### Statistical summary

**Figure 2:**
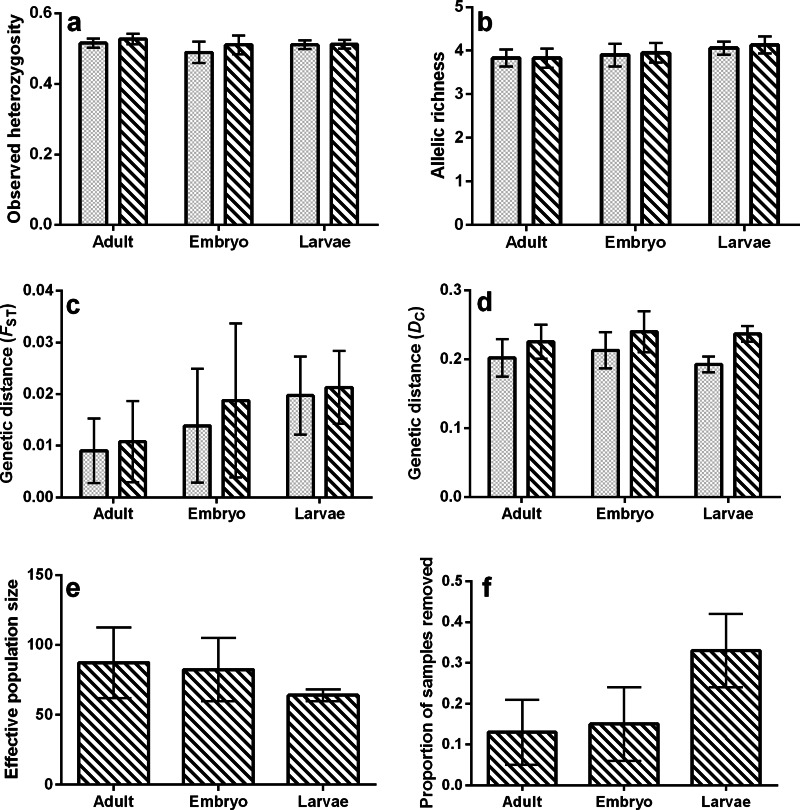
Bar plots representing mean values of (A) observed heterozygosity, (B) rarefied allelic richness (*A*_*r*_), (C) genetic distance (*F*_ST_), (D) genetic distance (*D*_*C*_), (E) effective population size (*N*_*e*_), and (F) proportion of samples removed due to sibship. Solid bars represent values containing full siblings, patterned bars represent values after sibling removal, and error bars represent standard deviations.

For all tests, none of the population genetic parameters differed significantly among life stages (bootstrap ANOVA *P*-value > 0.05) or within ponds and among life stages (permutation *t*-test *P*-value ≥ 0.25; Fig. 2, Tables 1 and 2), regardless of whether or not siblings were present in the data. The proportion of samples removed due to sibship was nearly significant (bootstrap *P*-value = 0.053, Fig. 2F), with a greater proportion of field-collected samples being omitted from larvae. There was also an increase in the estimated mean *F*_ST_ calculated in the larval and embryo data as compared to adults (Fig. 2C), but we note that this increase was not significant. However, this trend was not observed when genetic distance was measured using allele frequencies (*D*_*C*_, Fig. 2D). Values of population genetic summary statistics calculated on data sets with siblings removed are given as the mean (±standard deviation). Effective population size among ponds averaged 87.4 (±25.28) for adults, 82.4 (±25.58) for embryos, and 64 (±4.32) for larvae. Average rarefied allelic richness of adults was 3.83 (±0.22), 3.95 (±0.23) for embryos, and 4.13 (±0.20) for larvae. The average observed heterozygosity was 0.53 (±0.01) for adults, 0.51 (±0.03) for embryos, and 0.51 (±0.01) for larvae. On average, we removed 33.2% (±0.09) of larval samples due to sibship, while only 13% (±0.084) of adult and 14.98% (±0.09) of embryo samples were removed. With siblings removed, pairwise genetic distances between ponds measured using *F*_ST_ averaged 0.011 (±0.008) in adults, 0.021 (±0.007) in larvae, and 0.019 (±0.014) in embryos, while *D*_*C*_ averaged 0.226 (±0.025) in adults, 0.237 (±0.011) in larvae, and 0.240 (±0.030) in embryos (Table 3).

**Table 1:** Summary statistics following COLONY analysis including effective population size (*N*_*e*_) and the proportion of samples removed. The combined life stages sibling removal follows two iterations of COLONY, the first within life stage, the second after pooling life stages.

	*N*_*e*_			Proportion of samples removed			
Pond	Adult	Embryo	Larvae	Adult	Embryo	Larvae	Combined life stages
1	120	92	62	0.08	0.08	0.21	0.40
2	67	114	–	0.16	0.05	–	0.25
3	100	93	–	0.00	0.12	–	0.24
4	100	55	70	0.16	0.20	0.39	0.41
5	50	58	60	0.25	0.30	0.40	0.37
Avg	87.4	82.4	64	0.13	0.15	0.33	0.33
SD	25.28	22.58	4.32	0.08	0.09	0.09	0.07

**Table 2:** Rarefied allelic richness and observed heterozygosity estimates at each pond for both the full data set, and with siblings removed. Mixed-tissue is the bootstrap mean and 95% confidence interval from randomly sampling all life stages together. Bolded type indicates values that fall outside of the bootstrapped 95% confidence interval.

	*A*_*r*_				*H*_*o*_			
Pond	Adult	Embryo	Larvae	Mixed samples (95% CI)	Adult	Embryo	Larvae	Mixed samples (95% CI)
*Full data set*								
1	4.11	4.22	4.21	4.22 (3.99–4.44)	0.54	0.49	0.52	0.53 (0.49–0.57)
2	3.94	4.10	–	4.08 (3.86–4.27)	0.51	**0.45**	–	0.50 (0.47–0.53)
3	3.65	3.68	–	3.67 (3.44–3.89)	0.50	0.47	–	0.49 (0.46–0.53)
4	3.77	3.63	3.90	3.73 (3.50–3.98)	0.52	0.50	0.49	0.51 (0.47–0.54)
5	3.66	3.88	4.07	3.92 (3.64–4.17)	0.50	0.54	0.52	0.54 (0.50–0.57)
Avg	3.83	3.90	4.06	3.93 (3.51–4.35)	0.52	0.49	0.51	0.52 (0.48–0.56)
SD	0.20	0.26	0.15	–	0.01	0.03	0.02	–
*Siblings removed*								
1	4.16	4.20	4.36	4.22 (4.00–4.41)	0.55	0.53	0.51	0.54 (0.50–0.57)
2	3.98	4.15	–	4.19 (4.05–4.31)	0.52	**0.48**	–	0.51 (0.49–0.53)
3	3.65	3.72	–	3.77 (3.57–3.95)	0.50	0.48	–	0.50 (0.47–0.53)
4	3.77	3.64	3.88	3.81 (3.57–4.03)	0.54	0.52	0.50	0.52 (0.49–0.56)
5	3.57	4.04	4.16	3.98 (3.71–4.21)	0.53	0.54	0.53	0.54 (0.51–0.57)
Avg	3.83	3.95	4.13	3.99 (3.62–4.35)	0.53	0.51	0.51	0.52 (0.48–0.56)
SD	0.22	0.23	0.20	–	0.01	0.03	0.01	–

**Table 3:** Pairwise genetic distances (*F*_ST_ and *D*_*C*_) between ponds for each life stage and for mixed-tissue life stages. Mixed is the bootstrap mean and 95% confidence interval from randomly sampling all life stages together. Bolded type indicates values that fall outside of the bootstrapped 95% confidence interval.

	*F*_ST_				*D*_*C*_			
Pond-pair	Adult	Embryo	Larvae	Mixed (95% CI)	Adult	Embryo	Larvae	Mixed (95% CI)
*Full data set*								
1_2	0.000	0.000	–	0.003 (−0.006–0.014)	0.168	0.195	–	0.190 (0.163–0.216)
1_3	0.012	0.002	–	0.011 (0.002–0.023)	0.212	0.208	–	0.235 (0.206–0.267)
1_4	0.019	0.020	0.020	0.008 (−0.001–0.020)	0.205	0.247	**0.193**	0.237 (0.204–0.268)
1_5	0.015	0.024	0.029	0.022 (0.008–0.037)	0.227	0.250	**0.204**	0.237 (0.208–0.267)
2_3	**0.005**	**0.006**	–	0.019 (0.007–0.032)	0.196	0.201	–	0.209 (0.183–0.237)
2_4	0.011	**0.023**	–	0.004 (−0.006–0.017)	**0.188**	0.230	–	0.218 (0.192–0.244)
2_5	0.013	0.030	–	0.022 (0.008–0.038)	0.216	0.233	–	0.211 (0.183–0.240)
3_4	**0.000**	**0.004**	–	0.019 (0.007–0.032)	0.151	0.165	–	0.171 (0.142–0.200)
3_5	0.012	**0.023**	–	0.010 (0.000–0.022)	0.242	0.207	–	0.221 (0.192–0.250)
4_5	0.005	0.012	0.011	0.010 (0.000–0.023)	0.213	0.197	**0.181**	0.223 (0.194–0.252)
Avg	0.009	0.014	0.020	0.013 (0.002–0.026)	0.202	0.213	0.193	0.215 (0.187–0.244)
SD	0.006	0.011	0.009	–	0.027	0.027	0.011	–
*Siblings removed*								
1_2	0.000	0.000	–	0.002 (−0.005–0.011)	0.191	0.199	–	0.187 (0.164–0.212)
1_3	0.015	**0.001**	–	0.011 (0.003–0.020)	0.240	0.218	–	0.230 (0.206–0.257)
1_4	**0.024**	**0.021**	**0.024**	0.008 (0.000–0.017)	0.243	**0.258**	0.234	0.229 (0.200–0.256)
1_5	0.020	**0.036**	0.028	0.017 (0.006–0.030)	0.242	**0.273**	0.249	0.224 (0.195–0.251)
2_3	**0.005**	0.010	–	0.016 (0.007–0.027)	0.222	0.233	–	0.214 (0.193–0.234)
2_4	0.013	0.023	–	0.006 (−0.004–0.018)	0.220	**0.259**	–	0.213 (0.189–0.235)
2_5	0.015	**0.040**	–	0.019 (0.007–0.031)	0.226	**0.276**	–	0.203 (0.178–0.228)
3_4	**0.000**	0.006	–	0.016 (0.007–0.028)	0.179	0.191	–	0.173 (0.146–0.203)
3_5	0.013	**0.037**	–	0.007 (−0.001–0.017)	**0.262**	**0.256**	–	0.212 (0.189–0.237)
4_5	0.004	0.020	0.012	0.008 (−0.002–0.020)	0.232	**0.240**	0.227	0.213 (0.187–0.239)
Avg	0.011	0.019	0.021	0.011 (0.002–0.022)	0.226	**0.240**	**0.237**	0.210 (0.185–0.235)
SD	0.008	0.014	0.007	–	0.025	0.030	0.011	–

**Figure 3:**
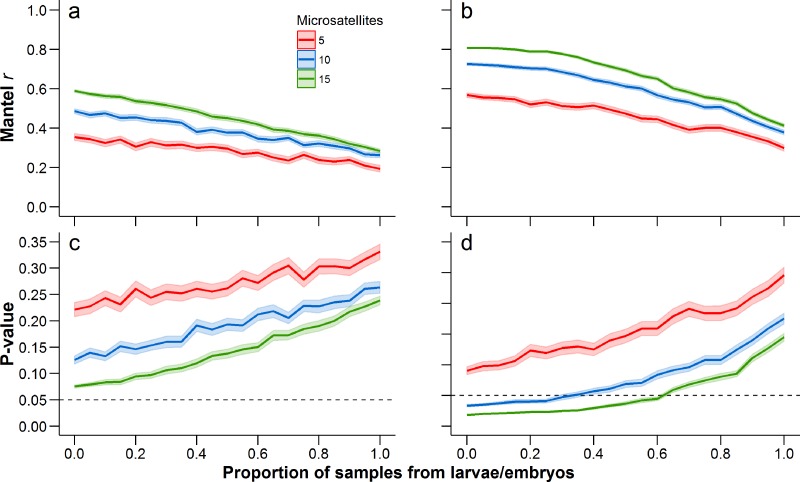
Change in Mantel *r* when using *F*_ST_ (A) and *D*_*C*_ (B), and the corresponding change in the *P*-value (*c* = *F*_ST_; *d* = *D*_*C*_) with increasing proportion of tissue samples coming from larvae and embryos. The dashed line in (C) and (D) is drawn at 0.05 to indicate the traditional threshold for significance. Mean (solid line) and 95% confidence intervals (lighter shading) were estimated at 0.05 increments between from 0 to 1. A proportion of 0 represents an adult-only sample, while a proportion of 1 represents a larvae/embryo-only sample. At each 0.05 increment, 1,000 bootstrap samples were conducted and Mantel *P*-values were estimated from 100,000 permutations. Each of these statistics was calculated with 5, 10, and 15 microsatellites (full data set), with a different set of microsatellites being randomly selected at each bootstrap iteration.

When comparing each life stage at each summary metric, we found no significant differences between data containing the sibling pairs and data with removed sibling pairs (permutation *t*-test *P*-value ≥ 0.19; Fig. 2, Tables 1 and 2). We found that the mixing of life stages resulted in genetic estimates of *A*_*r*_, *H*_*o*_, *F*_ST_, and *D*_*C*_ that, on average, did not differ from estimates made for each specific life stage (Tables 1 and 2). There were, however, up to three pond-pair *F*_ST_ values that fell outside of the bootstrapped 95% confidence intervals (Table 3). When genetic distance was measured using *D*_*C*_, only one pond-pair fell outside of the bootstrapped confidence interval. In general, there was a greater frequency of pairwise genetic distance measures based on embryo samples to fall outside of the mixed sample confidence interval. For both *F*_ST_ and *D*_*C*_, the removal of siblings resulted in more pairwise estimates falling outside of the mixed sample confidence interval (Table 3). Due to sample size, clear inferences cannot be drawn from larvae. With regard to IBD, only tests using adult samples (with and without siblings) resulted in significant relationships (Table 4), and the IBD relationship was stronger when genetic distance was measured using *D*_*C*_ (Fig. 3). IBD tests from embryos or larvae had much lower Mantel *r* correlations and were not significant. The mixing of tissue samples resulted in non-significant IBD tests when using *F*_ST_ (*p* > 0.05; Table 4), but had little effect when using *D*_*C*_ (Table 4).

**Table 4:** Results of simple Mantel tests assessing the correlation between genetic distance and geographic distance. Mixed life stage represents 1,000 bootstrap iterations, and the corresponding Mantel *r* and *P*-value estimates are the mean and 95% confidence intervals of the bootstrap iterations. Mantel *P*-values were estimated from 100,000 permutations.

	*F*_ST_		*D*_*C*_	
Life stage	Mantel *r*	*P*-value	Mantel *r*	*P*-value
*Full data set*				
Mixed	0.38 (−0.04–0.71)	0.18 (0.03–0.54)	0.775 (0.505–0.943)	0.031 (0.016–0.100)
Adult	0.715	0.034	0.731	0.033
Embryo	0.164	0.316	0.687	0.033
Larva	−0.125	0.666	−0.055	0.668
*Siblings removed*				
Mixed	0.310 (−0.10–0.65)	0.22 (0.05–0.54)	0.758 (0.467–0.936)	0.035 (0.016–0.100)
Adult	0.704	0.033	0.794	0.016
Embryo	0.093	0.417	0.427	0.118
Larva	0.190	0.667	−0.186	0.667

Our assessment of increasing the proportion of larval or embryo tissue samples clearly demonstrated that the calculated Mantel *r* decreases as the proportion of larval and embryo samples increases (Fig. 3). This pattern was consistent regardless of whether genetic distance was measured using *F*_ST_ or *D*_*C*_. Correspondingly, the average *P*-value of the Mantel test increased as the proportion of larval and embryo samples increased. Further, reducing the number of microsatellites resulted in a reduced Mantel correlation and increased *P*-value (Fig. 3). When using all 15 microsatellites for this analysis, the mean bootstrapped *P*-value for tests using *F*_ST_ started at 0.057 (0.055–0.60) for adult-only samples, and increased to 0.231 (0.223–0.239) for larvae/embryo-only samples (Fig. 3B). In contrast, the *P*-value for Mantel tests with *D*_*C*_ started at 0.019 (0.018–0.020) when only adults were included and increased to 0.146 (0.140–0.152) without adult samples (Fig. 3D). The 0.05 *P*-value threshold is passed when the proportion of larvae and embryos in the sample reaches 0.60–0.65 (*P*-values = 0.047–0.054, respectively). When 10 microsatellites are used, the 0.05 threshold is exceeded when the proportion of larvae and embryos reaches 0.30–0.35 (*P*-values = 0.047–0.052, respectively). The IBD relationship was not significant at any mixture proportion when only five microsatellites were used.

**Figure 4:**
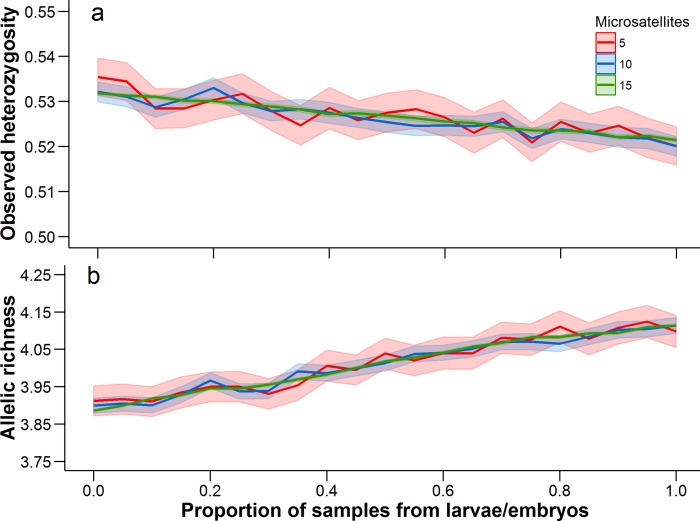
Observed heterozygosity and allelic richness, averaged over all populations. Mean (solid line) and 95% confidence intervals (lighter shading) were estimated at 0.05 increments between from 0 to 1. A proportion of 0 represents an adult-only sample, while a proportion of 1 represents a larvae/embryo-only sample. At each 0.05 increment, 1,000 bootstrap samples were conducted. Each of these statistics was calculated with 5, 10, and 15 microsatellites (full data set), with a different set of microsatellites being randomly selected at each bootstrap iteration.

There was a trend for the mean heterozygosity to decrease and mean allelic richness to increase as the proportion of larvae and embryos increased, but we note that these trends occur over a very limited parameter space (Fig. 4). Adult-only samples had a mean observed heterozygosity of 0.533 and mean allelic richness of 3.89, while larvae/embryo-only samples had a mean observed heterozygosity of 0.521 and mean allelic richness of 4.11. The number of microsatellites used in the analysis did not affect the mean estimate of heterozygosity or allelic richness, although precision in the bootstrap estimates was greater with more microsatellites.

## Discussion

Our literature search revealed that mixed tissues have been used in about one fifth of amphibian population genetic studies using microsatellites, despite the lack of knowledge concerning the effects that this may have on population or landscape genetic inferences. Collection of genetic samples from the field is likely influenced by numerous factors, such as the timing of life stage development and accessibility to sampling sites. Many of the studies that mixed tissues did so when the target life stage was not found and minimum sample requirements had to be met. Decisions made during sampling can undoubtedly influence the inferences made, as genetic variation within structured populations can vary spatially, temporally, or as a result of life history (Anderson & Dunham, 2008; Schwartz & McKelvey, 2009; Scillitani et al., 1996).

In our study, however, we found little evidence for adverse effects of including siblings or with mixing tissue samples collected from different life stages when assessing levels of allelic richness, heterozygosity, or effective population size. Contrary to Goldberg & Waits (2010), it may not always be necessary to remove siblings from a dataset to achieve unbiased population genetic estimates. Nonetheless, we urge researchers to proceed with caution when there is a high likelihood that full-siblings are present in a data set, and to carefully assess the tradeoffs between power and precision. We found that estimates of pairwise *F*_ST_ differed substantially between life stages as well as from the mixed-tissue sample, but this difference was not statistically significant. Additionally, this pattern was not observed when pairwise genetic distance was measured using *D*_*C*_. The greatest impact of mixing life stages was evident in our tests of IBD among pond pairs. Only the tests using adults were significant, while larvae-only, embryo-only, and mixed-tissue samples showed little correlation with distance. The IBD relationship was strongest when genetic distance was measured using *D*_*C*_, and we found that the IBD relationship seen in adult-only samples decreased as the proportion of larvae and embryos included in the population sample increased. In our dataset, the IBD relationship, when assessed with *D*_*C*_, remained significant until the frequency of larvae and embryos in the population sample exceeded 60%. However, our power to detect a significant IBD relationship was substantially affected by the number of microsatellites used. When 10 of the 15 microsatellites were used, the IBD relationship became non-significant when the frequency of larvae and embryos exceeded 30%, and no significant IBD relationship was evident when only five microsatellites were used. As such, our results suggest the greatest effects of mixing different life stages may be evident in landscape genetic analyses assessing pairwise distances among populations, with different conclusions potentially being drawn from adult-only samples as compared to larvae, embryo, or mixed-tissue samples. Further, such relationships may be sensitive to the genetic distance statistic used as well as the number or polymorphism of the microsatellites used.

Contrary to our predictions, we did not observe significant biases in our population genetic measures among life stages or with the exclusion of full siblings from the data. This is counter to the findings of Goldberg & Waits (2010) who found that skewed estimates between larval and adult population genetic measures were eliminated or reduced when full siblings were removed from the larval sample. As in the analysis of mixed-tissue samples, the greatest differences were observed in relation to genetic differentiation measured by *F*_ST_, which increased (insignificantly) after the removal of full siblings from the data, as well as from adults to embryos, to larvae. These increases in genetic differentiation in the embryonic and larval stages, as compared to the adults is predicted by population genetic theory (Allendorf & Phelps, 1981), and has been empirically demonstrated in Columbia spotted frogs (*Rana luteiventris*) (Goldberg & Waits, 2010).

The clearest result from our study was the proportion of samples that had to be removed from each life stage due to redundancy of siblings. It is actually quite unlikely that we sampled full sibling adults given their life history and longevity (Petranka, 1998), but we chose to remove these putative siblings from our data set for consistency of methods among life stages. We note, however, that Goldberg & Waits (2010) did not test for or remove sibling pairs from their adult samples. In our study, up to 40% of larval samples were found to be from sibling pairs, which would have to be removed if their inclusion biased genetic parameter estimates. Although larvae are often the most readily accessible and conveniently sampled life stage, this represents an inefficient use of resources. When possible, it may be prudent to avoid sampling larvae. Ultimately, the choice of which life stage or stages to sample will be idiosyncratic to the study and system. We have demonstrated that inferences differed minimally and insignificantly between larvae, embryos, and adults, but we note that both larvae and adults can be sampled non-destructively, which may become a factor in deciding which life stage to sample for some species.

In our study, we assessed IBD through the use of simple Mantel tests. We readily acknowledge the limitations and criticisms of the Mantel test for making robust inference (e.g., Guillot & Rousset, 2013; Legendre, Fortin & Borcard, 2015). However, we feel that for our limited data set and ultimate goal of assessing relative differences and patterns between life stages, mixed life stage samples, and genetic distance measures, the simple Mantel test was sufficient and provides an appropriate cautionary caveat for future researchers using any method. More rigorous methods such as distance-based redundancy analysis (Legendre & Anderson, 1999), multiple regression of distance matrices (Holzhauer et al., 2006), distance-based Moran’s eigenvector maps (Legendre & Legendre, 2012), or mixed effects models fit with an appropriate error structure (Clarke, Rothery & Raybould, 2002) should preferentially be used over Mantel tests in future studies seeking to estimate the effects of landscape features on genetic differentiation.

To our knowledge, our study is the first attempt to determine how the sampling and mixing of different life stages affects genetic parameter estimates. It is unclear how differences in life history (e.g., life span, breeding site fidelity, reproductive strategy, etc.) alter the effects of sampling different life stages, as we currently do not have a mechanistic explanation for the patterns we observed. We found that mixed-tissue samples can lead to different conclusions when conducting spatial analyses, such as IBD, and these results would likely extend to more complex landscape genetic analyses as well. As such, we caution researchers to carefully consider the implications of mixing samples collected from multiple life stages. Our finding that population genetic parameters differed little with the removal of siblings or mixing of life stages was surprising and contrary to our predictions. In our study, we had a relatively small sample size from which to draw inference, although we note that it is not much smaller than that used by Goldberg & Waits (2010) who assessed eight populations of *R. luteiventris* and four populations of *A. macrodactylum*. We do note that the power to infer differences is likely greater in our study due to the large number of polymorphic microsatellites used, which was reinforced by our results of subsampling the number of genetic markers used. Perhaps of greater importance is the fact that our populations are relatively close together (maximum distance = 3,200 m) and situated within continuous forest habitat. In contrast, Goldberg & Waits (2010) sampled populations separated by 2.7–18.5 km of agricultural matrix. Further, the populations included in our study are very robust, with recorded breeding aggregations of several hundred individuals (R Semlitsch, 2009, unpublished data).

Like Goldberg & Waits (2010), we suggest that future studies include a pilot phase to assess the effects of sampling different life stages to meet the objectives of the specific project. An important consideration for any population or landscape genetic study is the target demographic group for which inferences are desired. If quantifying movement or connectivity of populations is the main objective of a study, then sampling adult life stages may provide the most accurate inferences. If the study objectives are to quantify the distribution of genetic diversity, then sampling of embryos appears to be the most efficient use of resources. However, nearly equivalent estimates can be obtained from larvae, and as demonstrated in this study, the presence of siblings may not result in biased estimates. Ultimately, the mechanisms underlying the patterns observed in this study are not known, but this may be a fruitful avenue for future research to explore through simulation modeling to better understand how variation life history characteristics and sampling scenarios affect population and landscape genetic inference.

## Supplemental Information

10.7717/peerj.1813/supp-1 Supplemental Information 1 Supplemental summary data tables

## Additional Information and Declarations

### Field Study Permissions

The following information was supplied relating to field study approvals (i.e., approving body and any reference numbers): Permits approved and issued by the Missouri Department of Conservation. Missouri Wildlife Collector’s permit 15584.

### Data Availability

The following information was supplied regarding data availability: Figshare: https://dx.doi.org/10.6084/m9.figshare.1621318.
