# Supplementary material for: Reducing bias in population and landscape genetic inferences: the effects of sampling related individuals and multiple life stages
Source: PeerJ. 2016 Mar 14;4:e1813. doi: 10.7717/peerj.1813 (PMC4793335; doi:10.7717/peerj.1813)
Supplement: Supplemental Information 1 [file peerj-04-1813-s001.docx]

**Supplement** **1.** Number of samples collected per pond. Values in parentheses are the number of samples remaining after the removal of full siblings.

| **Pond** | **Adults** | **Embryos** | **Larvae** | **Combined life stages** | |
| --- | --- | --- | --- | --- | --- |
| 1 | 25 (23) | 24 (22) | 29 (23) | 78 (47) |  |
| 2 | 25 (21) | 19 (18) | – | 44 (33) |  |
| 3 | 25 (25) | 26 (23) | – | 51 (39) |  |
| 4 | 25 (21) | 25 (20) | 36 (22) | 86 (51) |  |
| 5 | 24 (18) | 27 (19) | 35 (21) | 86 (54) |  |
| Avg | 24.8 (21.6) | 24.2 (20.4) | 33.33 (22) | 69 (44.8) |  |
| SD | 0.45 (2.61) | 3.11 (2.07) | 3.79 (1.00) | 20.05 (8.67) |  |

**Supplement 2**. Summary statistics for microsatellite loci used in this study including number of individuals for which the microsatellite amplified (N), total number of alleles per locus (N_a_), observed heterozygosity (H_o_), and expected heterozygosity (H_e_).

| **Locus** | **N** | **N_a_** | **H_o_** | **H_e_** | **P-value** |
| --- | --- | --- | --- | --- | --- |
| Am_56 | 279 | 3 | 0.419 | 0.425 | 0.885 |
| Am_34 | 279 | 9 | 0.731 | 0.805 | 0.049 |
| Am_62 | 279 | 3 | 0.355 | 0.381 | 0.142 |
| Am_4 | 279 | 7 | 0.215 | 0.222 | 0.209 |
| Am_9 | 279 | 4 | 0.516 | 0.507 | 0.663 |
| Am_39 | 279 | 4 | 0.559 | 0.521 | 0.617 |
| Am_21 | 279 | 5 | 0.38 | 0.41 | 0.058 |
| Am_10 | 279 | 7 | 0.649 | 0.682 | 0.165 |
| Am_29 | 279 | 4 | 0.161 | 0.174 | 0.057 |
| Am_30 | 278 | 6 | 0.683 | 0.706 | 0.439 |
| Am_38 | 277 | 3 | 0.542 | 0.523 | 0.061 |
| Am_3 | 277 | 14 | 0.784 | 0.817 | 0.053 |
| Am_55 | 279 | 3 | 0.452 | 0.477 | 0.349 |
| Am_7 | 279 | 7 | 0.667 | 0.628 | 0.222 |
| Am_37 | 277 | 5 | 0.7 | 0.705 | 0.058 |
